# Influence of psychological autonomy support of peer instruction: A novel interactive approach using Instagram in language learning

**DOI:** 10.3389/fpsyg.2022.866354

**Published:** 2022-09-20

**Authors:** Hind Alfadda, Muhammad Afzaal, Hassan Mahdi, Rasha Alaudan, Samantha Curle

**Affiliations:** ^1^King Saud University, Riyadh, Saudi Arabia; ^2^Shanghai International Studies University, Shanghai, China; ^3^Department of Education, University of Bath, Bath, United Kingdom

**Keywords:** Just-in-Time, peer instruction, psychological motivation, language acquisition, Instagram and, Snapchat stories

## Abstract

This study makes an original contribution to knowledge by investigating the impact of Just-in-Time (JiT) teaching and peer instruction (PI) strategies on the promotion of students’ achievement, interaction, and motivation in English language learning. Students were recruited from an undergraduate TESOL program in the first semester of 2019 at a University in Saudi Arabia. A multiple method research design was used to address the research questions robustly and rigorously. First, a two-group quasi-experimental design was implemented. In the first group, a lecture-based strategy was used (*n* = 28), while in the second group, JiT and PI strategies were used (*n* = 27) in English language lessons. An innovative research method was used in this study: a private Instagram account was created, serving as a platform through which teacher and students could communicate using interactive posts and comments. Second, students filled out a survey reflecting on this medium of language teaching and learning. Finally, focus group interviews were conducted to determine students’ views on the use of Instagram as an educational tool in terms of its flexibility and usefulness. The findings of the study suggest that JiT teaching, and PI promoted students’ achievement and enhanced students’ motivation, particularly in relation to fluency and novelty in the second language. Furthermore, students viewed Instagram as a successful educational tool that significantly facilitated their collaboration. Nevertheless, this study also highlights the challenges of using JiT teaching and PI to facilitate language learning. Suggestions for practitioners are specified; including providing adequate e-learning tools for students to enhance language learning motivation and achievement.

## Introduction

Technology plays a vital role in the teaching and learning process. It facilitates and adds to the excitement of the lesson in many ways. Important factors that technology can enhance include motivation and collaboration through social media, as suggested by Al-Adwani and Al-Fadley (2017). Motivation is mainly associated with activating students’ background knowledge. This study examines Just-in-Time (JiT) teaching and peer instruction (PI) as effective instructional strategies in language teaching. These strategies help classroom teachers receive feedback from students, evaluate students’ learning progress through reviews of their online learning performance and tailor lesson plans accordingly. JiT teaching requires students to complete online assignments, such as reading and watching videos before class. On the other hand, PI provides students with opportunities to collaborate in classroom discussions to clarify any misunderstandings of the topic with the assistance of the classroom teacher.

This study makes an original contribution to knowledge by combining the JiT teaching strategy with PI using the Instagram application as a tool to enhance collaboration and increase student motivation and language learning outcomes. The JiT strategy and PI with the use of Instagram can be beneficial to English language learning for the following reasons: (a) increased collaboration and discussion among students inside and outside of the classroom, (b) taking a tailored approach to students’ different learning styles, and (c) ease and enjoyability of use. To date, no such study has been conducted, this study will therefore fill this knowledge gap.

The following research questions were addressed in this study:

1. Does the use of Just-in-Time teaching, and peer instruction enhance students’ achievement in a second language?
2. What are students’ perceptions of Instagram to enhance discussion, collaboration, and motivation?
3. What are the relationships between using Instagram in pre-class teaching, active learning, reflection, and usability and flexibility of Instagram in second language learning?

## Literature review

### Effective English as a foreign language teaching with technology

The use of technology in teaching and learning English as a second language has recently become more common place. Using technology in language teaching and learning provides numerous opportunities for language teachers and learners. Technology offers many resources and tools for language teachers that can be used to create positive environments for language learning. Language learners can be exposed to authentic language input, and they can practice the language in a creative way. Ghanizadeh et al. (2015) found that technology can be used in almost all areas of language learning. Technology was useful in enhancing the quality of input, making communication real, and providing relevant feedback. The use of technology has therefore become nearly ubiquitous in language teaching and learning (Shadiev and Yang, 2020). New technologies such as virtual reality and augmented reality have been developed, and increasingly becoming available to the general public, as well as language practitioners (Shadiev et al., 2019). Moreover, emerging technologies (e.g., cloud computing and natural language processing) are expanding, showing to be very promising when used for language learning and teaching. Studies have shown that technology can stimulate the learners’ performance, boost motivation and provide learners with more effective ways of language learning (Shadiev and Huang, 2020). This study aims to make an original contribution to the field of technology-use for language teaching and learning.

In a study in Middleton and Murray (1999), found that students who used technology regularly in the classroom scored better than those who did not. With the integration of technology, the learners’ confidence and ability are likely to increase due to them being able to learn using their preferred method, thereby enhancing learning outcomes (Lee and Vail, 2004). Findings from Fouz-González (2020) also supports this argument: they found that the use of the English File Pronunciation (EFP) Application helped foreign language learners to improve their pronunciation. Besides Apps, social media is fast becoming an effective and essential part of technology that is considered an effective tool for teaching and learning language (Tess, 2013; Manca and Ranieri, 2016).

#### Social media

Technology used for language learning can take the form of using many tools and platforms. For example, multimedia and social media. Social media, however, were originally designed for communication, not for education, but can also be used for language learning. Social media provides language learners with the opportunity to share ideas and discuss many issues in the target language, permitting the development of students’ language and literacy (Beauvois, 1992; Chun, 1994). The proliferation of social media is due to their various forms, which help people communicate in various ways: such as blogs, wikis, video podcasts, and photo sharing (Slim and Hafedh, 2019). Applications such as Facebook, Twitter, Instagram, Little Red Book, WeiBo, YouTube, and WhatsApp have massively boosted social interaction and information sharing within student and teacher communities alike.

Social media has also been shown to help shy learners express themselves in the target language, more than they usually would in class (Kroonenberg, 1995). It also benefits educators in creating more student-centered, dynamic lessons compared to traditional methods of teaching (Bebell and Kay, 2010: 17). Research has also shown that learners prefer to engage in discussion online than face-to-face (see Sullivan and Pratt, 1996; Warschauer, 1996). Fifteen years ago, Thorne and Black (2007: 134) already noted the increase in second language (L2) learners using online resources to learn the target language, often substituting social media for traditional paper periodicals. Social media is a familiar environment for students: an interface though which they post content and communicate with peers (Zvarych et al., 2020). The social media platform of Instagram has therefore been used in this study.

#### An overview of using Instagram in the EFL classroom

Instagram is a new tool that is increasingly being used for learning a second language. It is considered an effective educational tool specifically for college students as it provides a platform for students to be creative with language (Kirst, 2016). Handayani (2018) notes how Instagram provides the opportunity for teachers and learners to communicate and engage in discussion about learning materials outside the formal classroom learning environment. Handayani (2018) emphasizes that Instagram is an “innovative tool” that needs to be used in education. After YouTube and Facebook, Instagram is the most widely used media platform in education (Al Fadda, 2020). Although numerous studies have been conducted on the use of Twitter and Facebook in teaching and learning, little research has been conducted on the use of Instagram and its effect on pedagogy, particularly in the Middle East. This study aims to fill this gap in knowledge.

A recent study conducted by using Instagram for language learning indicated that previous knowledge is essential, and that the recognition of text plays a crucial role in this learning process. Another study in Turkey conducted by Gonulal (2019) found that Instagram helped learners develop their language skills and that students were satisfied with the Instagram learning experience. It also showed that students’ use of Instagram helped them build their vocabulary and communication skills in English language learning. AlGhamdi (2018) also conducted a study using Instagram to develop students’ speaking and writing skills, and found an increase in skill development, and that students enjoyed this experience and were encouraged to be more creative. Table 1 presents further advantages (and disadvantages) of using Instagram in the English language learning classroom. In a study by Al Fadda (2020), English language learners reportedly preferred to use Instagram as a vehicle for language learning when lessons focused on simple (rather than complex) linguistic structures. Students also noted the ease and usability of Instagram. However, one disadvantage was the spelling and grammatical errors in this informal setting of language use. To overcome issues in relation to privacy, this study created a private Instagram account, accessible only by the students and language instructor.

**Table 1:** Perceived advantages and disadvantages of using Instagram to learn English.

	Advantages	Disadvantages
1	Readily available.	Lack of privacy.
2	Affordable.	Academic use may inhibit personal use.
3	Allows one to become immersed in an English language environment.	The information posted on Instagram may contain spelling and grammatical errors.
4	Provides an informal learning context.	Learning experiences based on Instagram may be too informal or unstructured.
5	Provides an opportunity to use the language in an authentic manner.	
6	Increases interest in learning English.	
7	Increases motivation to read English materials.	

Adapted from Aloraini and Cardoso (2018); Gonulal (2019); Yeh and Mitric (2019).

The current study makes a unique contribution to this growing body of literature by using Instagram as the main educational tool, but also incorporates JiT teaching and peer interaction (PI) into the language learning process.

### An overview of JiT teaching and PI

Just-in-Time teaching is a teaching strategy that was first implemented in the late 1990s. This strategy was designed to meet students’ specific learning needs. It is a pedagogical strategy that draws on feedback from students on activities done outside the classroom (e.g., at home or in this study, online), in preparation for class. During-class teaching and learning is therefore much more tailored to students’ current knowledge and gaps in knowledge. The main aims are to: maximize classroom teaching and learning time, increase student motivation, encourage students to engage with content outside of class time, and to provide the opportunity for instructors to adapt their teaching in line with students’ immediate needs.

In JiT teaching (JiTT), learners are provided with an opportunity to do an assignment before coming to class. This assignment is based on the content to be taught in the upcoming lesson. The impact of JiTT on teaching and learning has been examined across several academic disciplines (Simkins and Maier, 2010). The results of studies from different disciplines have shown statistically significantly higher learning outcomes, and a better understanding of academic concepts using JiT teaching (Riskowski, 2015). However, a dearth of studies have investigated the factors affecting the implementation of JiT in language teaching and learning (Flavián-Blanco et al., 2011; Casaló et al., 2021). This study is therefore innovative as it will explore the factors that affect the implementation of JiT teaching in English language learning.

Peer Interaction (PI) is a teaching technique that helps teachers promote student-to-student interaction *within* the class. Teachers pose questions to students that challenge their thinking to promote discussion and debate. PI allows students to discuss and participate in class, engaging in discussion activities that allow them to share their opinions and ideas, with confidence, in a friendly environment. Hoekstra (2008) states that students learn more in a relaxed, friendly environment. The use of Instagram in this study also aims to enhance this friendly learning environment.

Cross (1998) notes that PI nurtures a cooperative learning environment and therefore increases the chances of deeper learning among learners. Gokhale (1995) also advocates this element of cooperation suggesting that ‘explaining, justifying arguments, and reasoning’ help foster students’ critical thinking skills. Mazur (1997a) argues that this transcends academic discipline, and that PI (as well as JiT teaching) can be tailored to a variety of disciplines.

JiT teaching is an aspect of PI; facilitating the class using immediate feedback. Watkin and Mazur (2010) argue that JiT teaching works asynchronously out of class, and that PI gives real-time feedback. JiT teaching is a pre-class aspect of PI. Students complete online assignments, and the instructor then revises the online feedback from students and uses it to tailor the in-class discussion. In PI, students engage in group discussion and knowledge construction in class. Students then have the opportunity to teach each other through collaborative class activities such as group discussions and projects (Zou and Xie, 2019). Both PI and JiT teaching help teachers and students observe learning that takes place in the classroom and improve the teaching and learning process by improving the quality of feedback. These strategies also enhance opportunities to practice the English language with peers and teachers and over time enhance motivation to speak English.

### Factors that affect Just-in-Time teaching

Implementing JiT teaching is influenced by several factors. For example, pre-class assignments, that determine the nature of JiT teaching activities. Reflection, usability, and flexibility of technology also affect JiT teaching. These aspects will now be further elaborated.

#### Pre-class assignments and Just-in-Time teaching

Pre-class activities are useful to help students engage with content before they attend the class. Pre-class activities are useful for language learners in many ways. First, students obtain knowledge of the topic before engaging in in-class discussion. Second, students can prepare questions before learning about the topic in class. Pre-class learning is an important phase for learners to prepare for upcoming in-class activities (Mei, 2021). JiT teaching adopts pre-class assignments to foster these benefits.

#### Active learning and Just-in-Time teaching

Student engagement is key to successful teaching and learning, irrespective of the content and format of the content delivery mechanism (Khan et al., 2017). In a traditional, face-to-face classroom, many techniques can be used to make learning active. However, in an online learning environment (such as the switch to online learning during COVID-19), maintaining active learning might prove to be more challenging. To maintain active learning online, there are several strategies that can be used by instructors (see McDonald et al., 2020 for more detailed strategies). For example, using well-designed discussions and group work foster interaction. Just-in-Time teaching can also be used to foster active learning, as is the case in this study.

#### Reflection and Just-in-Time teaching

Students learn through reflection Hayes et al. (2015) pointed out that reflection is part of the learning process. Purposefully designed, in-line with learning outcomes and assessment, students should be given opportunities for reflection (Lock et al., 2017). Hayes et al. (2015) points out that whenever possible, teachers (particularly in an online environment) should ask learners to write a short reflection on their own performance, as assessed and evaluated against specified criteria. Students should then suggest action to improve performance and fill any knowledge gaps between their current performance and target performance. Reflection is a factor that is influenced by Just-in-Time teaching, as is measured in this study.

#### Usability and Just-in-Time teaching

The implementation of Just-in-Time teaching can also depend on the usability and flexibility of the tools used. Usability is understood as the measurement of how easy or challenging the interface is when used by a user (Nielsen, 2012). Rubin and Chisnell (2008) point out that any product can be accepted as “usable” if any user can find or achieve what they want without having problems or without requiring any help. Language instructors need to use technologies effectively in their teaching. To make Just-in-Time successful, the tools and platforms should be user-friendly. In this study, Instagram was deemed to be user-friendly and flexible in form and function.

### Theoretical framework

Both JiT teaching and peer instruction (PI) draw on *Constructivist Theory* (Kim, 2005). Constructivist theory suggests that learners actively construct knowledge rather than learn passively. All students come to class with some level of prior/background knowledge of the topic to be learned, and while learning new content, apply this background knowledge to construct new knowledge. This construction best takes place in an interactive learning environment where new ideas and information can be created (Guthrie et al., 2004). This study draws on this theory to explain how JiT teaching and PI assist in the language learning process.

## Methodology

This study employed a combined strategy of JiT teaching and PI with an experimental group of students. The JiT teaching component included a pre-class activity that was developed through web-based practice. Several types of activities were used in the pre-class activities. Laurel and Stephanie (2014) described activities involved in JIT teaching as “warm-ups” (designed to introduce new concepts and stimulate class discussion) and “puzzles” (designed to integrate various concepts to assess student learning following their working with the material).

### Data collection

Before data collection commenced all necessary ethical and legal permissions were obtained through the participating university. Informed consent was obtained from all participants, and all data collected was collected and stored in an anonymized format.

This study was then conducted over three months in the fall of 2019. The instructor structured the course so that a new topic (unit) was presented every week, with a total of 10 units completed over the entire three months. The 11th week lesson was used for revision, and the 12th week lesson used for the final exam.

**Figure 1:**
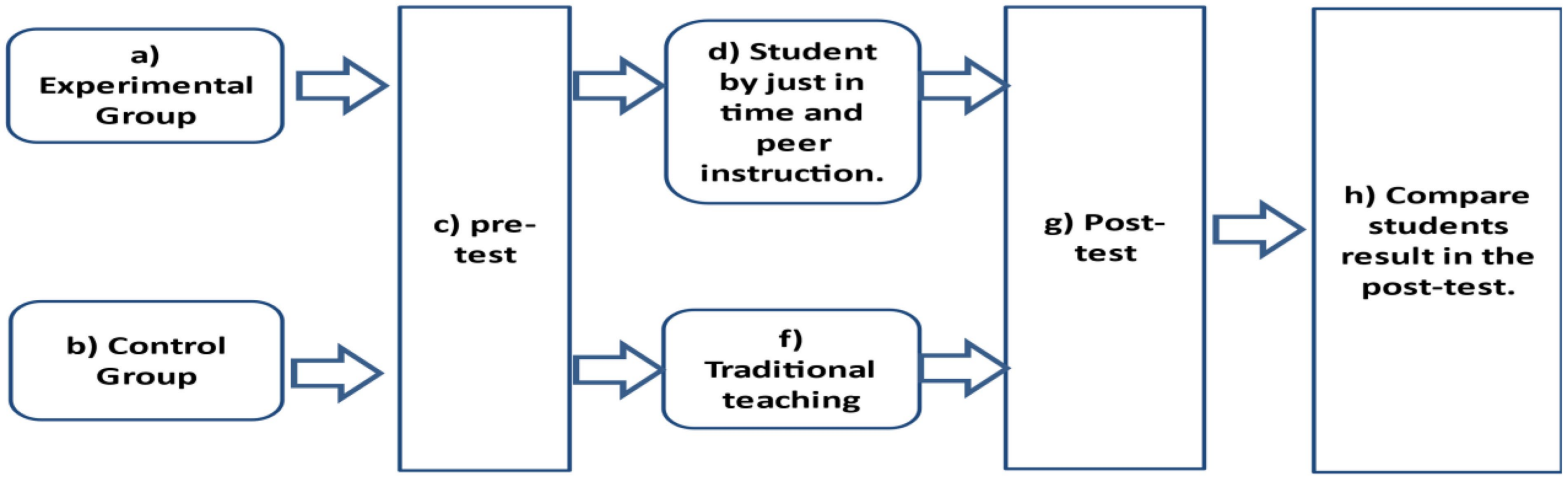
Stages of data collection.

To answer research question 1, the implementation of JiT teaching and PI was enhanced using Instagram as an assessment tool to enable students to interact with learning content by commenting on posts, and to allow teachers to track students’ progress. The instructor created a private Instagram account (@ nhg362class) and monitored students’ engagement on the Instagram page. Based on student interaction with the Instagram content, instruction in lessons were tailored to meet students’ learning needs. On the day of class, students were given a (pre)test with ten multiple-choice questions related to the previously posted materials. The time allocated to answer was 15 min. Based on students’ answers and engagement on the class Instagram page, the instructor determined what was confusing about the material and where students required additional support. Any confusing material was explained through collaborative group discussion PI in class with the instructor as the facilitator. At the end of the lesson, students were given a different set of questions to be answered in 10 min (post-test). Based on students’ progress, the instructor tailored the next lesson. In the control group, the instructor used the traditional method of teaching. On the day of the class, students were given a ten minute-pretest related to the new topic. The lesson then continued as normal. Students were then given a set of questions to be answered in 10 min (post-test). Both groups were given homework at the end of each lesson. Figure 1 illustrates the process of data collection.

In the experimental group, the instructor posted a video on the Instagram page @nhg362class one day before the class met. Students were required to log in to the Instagram class page to watch the video and answer the questions posted by the instructor under each video. The study used Instagram to develop spontaneous language skills; students were encouraged to use the language without feeling any pressure or fear of making mistakes. Instagram is very popular in Saudi Arabia, and the participants did not need any previous training to use the application. The students were very excited and eager to participate to use the application both inside and outside of the classroom. The participants did not receive any incentive for their participation. This quasi-experiment was part of the course’s normal activities in order to develop students’ language learning skills through the use of the social media application Instagram.

To answer research questions 2 and 3, a 21-item survey was given to students to collect data on their perceptions of the use of Instagram before and after class. This measured four dimensions of perception: pre-class teaching, active learning, reflection, and usability and flexibility of Instagram in second language learning.

#### Sample

This study focused on participants studying an undergraduate TESOL program at King Saud University (*n* = 55) in Saudi Arabia. This program provided students with the in-class flexibility to be fully active participants, not always the case in this context. The sample was divided into two groups: the experimental group (*n* = 27) and the control group (*n* = 28).

### Data analysis

In order to analyze the data, the Statistical Package for the Social Sciences (SPSS) version 22 was used to analyze the correlation between the categories of the questionnaire. In order to examine changes in learners’ performance from pre-test to post-test, a paired-samples t-test was used. To analyze the questionnaire, descriptive analysis was applied to determine the means and standard deviation of each item and construct in the questionnaire. Also, a Pearson correlation test was performed to explore the correlation between the constructs of the questionnaire. The values of Pearson correlations are summarized as follows, based on the benchmark proposed: small correlation = 0.10; medium correlation = 0.30 and large correlation = 0.50.

#### Reliability of the questionnaire

The questionnaire was carefully designed in accordance with the research questions, and the validity of the instrument ascertained. Cronbach Alpha was found to be reliable (*p* = 0.811), as shown in Table 2.

**Table 2:** Reliability statistics.

Cronbach’s Alpha	Cronbach’s Alpha based on standardized items	*N* of items
0.885	0.910	25

## Results

To answer the first research question, the researchers conducted a pre- and post-test for both groups at the same time. The pretest was given at the beginning of the semester; that is week 1. The test covered general information about the ten units and tested the students’ writing skills. The post-test was conducted at the end of week 12. A *t*-test was used to calculate the significance of the difference between the average pretest and post-test scores of the experimental and control groups; the results were as follows:

Tables 3, 4 indicate the equality of the two groups at the time of the pretest in terms of knowledge of language and information about topics that were presented throughout the course.

**Table 3:** Paired samples *t*-test for the control group.

Test	*N*	Mean	SD
Pretest	28	27.71	5.47
Post-test	28	28.34	4.91

Results of the *t*-test between the pretest scores (*M* = 27.71, SD = 5.47) and the post-test scores (*M* = 28.34, SD = 4.91); *t*(27) = 6.1093, *p* = 1.587^−6^. *p* < 0.05.

**Table 4:** Paired samples *t*-test for the experimental group.

Test	*N*	Mean	SD
Pretest	27	27.19	5.23
Post-test	27	38.44	4.89

Results of the *t*-test between the pretest scores (*M* = 27.19, SD = 5.23) and the post-test scores (*M* = 38.44, SD = 4.91); *t*(26) = 7.6588, *p* = 3.966^−8^. *p* < 0.05.

**Figure 2:**
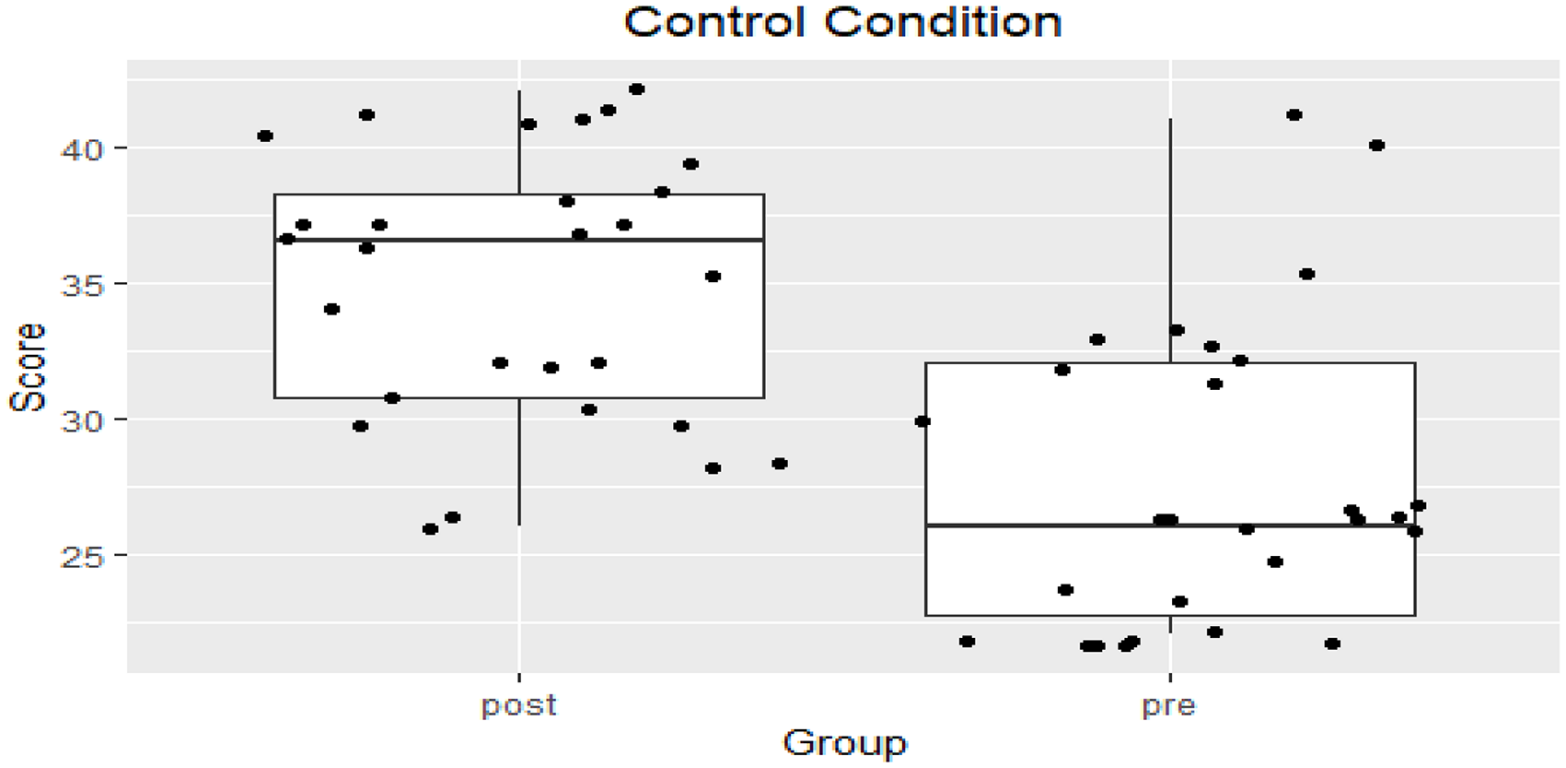
Control group pre- and post-test scores.

**Figure 3:**
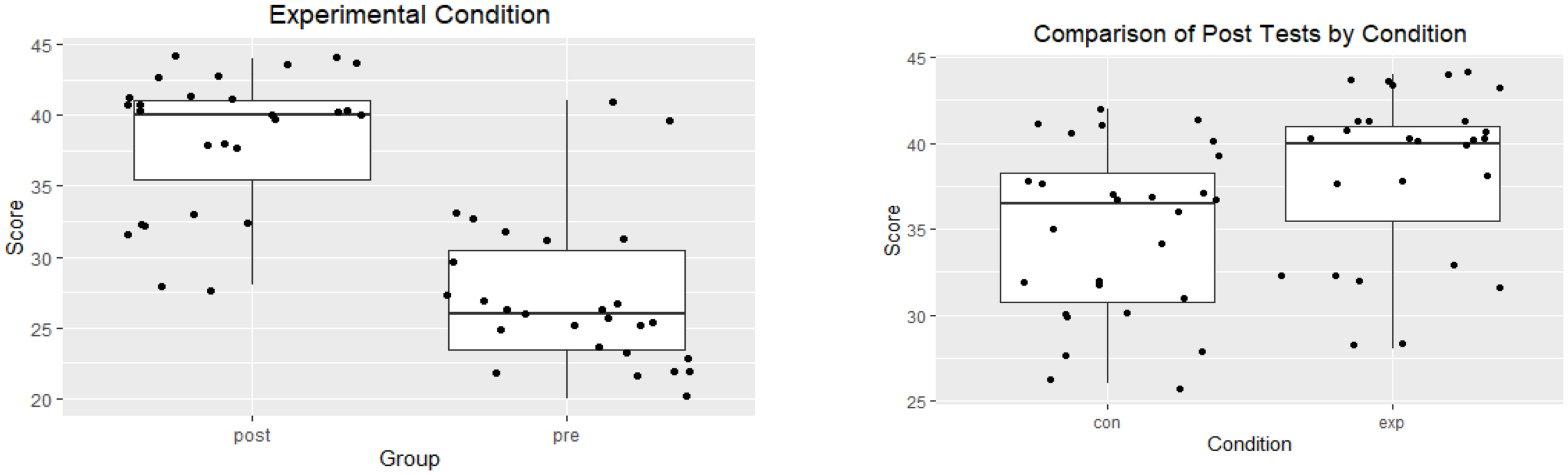
Comparison of control and experimental groups pre- and post-test scores.

To test the differences in academic achievement (see Figures 2, 3), the researchers referred to the achievement test scores of the experimental and control groups. The tests were administered at the end of week 12. A *t*-test was performed to find out the significance of the difference between the average post-test scores of the experimental and control groups; the results were as follows:

Results of the independent samples *t*-test between control conditions (*M* = 34.86, SD = 4.91) and the experimental conditions (*M* = 38.44, SD = 4.91); *t*(53) = −2.7083, *p* = 0.0090, *p* < 0.05.

Table 5 shows that the average of the post-test score of the experimental group reached 38.44, and that of the post-test of the control group reached 34.85. The results indicated better achievement of the experimental group (post-test). The results indicate a positive effect of the JiT and PI strategies on the level of academic achievement, as the differences in the results were in favor of the post-test scores of the experimental group.

**Table 5:** Independent samples t-test between conditions (control and experimental) post-test.

Conditions	*N*	Mean	SD
Control	28	34.86	4.91
Experimental	27	38.44	4.89

Figure 3 indicates that the JiT and PI strategies played a prominent role in enhancing students’ motivation to increase their levels of achievement and improve their performance and contribution in the class discussion, which led to a modification of students’ behavior. In addition, these strategies raised the level of student attention to the importance of skills to be learned and increased levels of interaction, collaboration, and motivation to learn skills in the NHJ365 course; these strategies helped students learn and helped the teacher address the weaknesses and shortcomings of the students.

To answer the second question of the study, a 21-item survey was distributed to students by four faculty members in the College of Education. The survey aimed to collect data regarding students’ perceptions of the use of Instagram before and after the class. The results of the survey are shown in Table 6.

**Table 6:** Statistical average of the responses regarding the use of Instagram.

Item	Statement	% agreement	Mean	SD
1	I love to read from Instagram rather than the book.	76%	3.21	0.82
2	Reading my friends’ comments has helped support my understanding.	93%	3.52	0.63
3	Reading my friends’ comments makes the topic more authentic.	93%	3.28	0.59
4	I would rather do my reading assignment from Instagram than from the book.	83%	3.48	0.74
5	I like to discuss my point of view with my friends in the comments section.	97%	3.45	0.57
6	I like to discuss the posted pictures in a written form rather than an oral form.	79%	3.93	0.68
7	My friends’ comments enhance my vision of the topic.	100%	3.41	0.50
8	I like to discuss our written comments in the classroom with my classmates.	100%	3.41	0.50
9	I enjoy debating opinions with my classmates in the comments section.	97%	3.38	0.56
10	Writing my opinions in the comments section helps me to think carefully about how to express my ideas clearly.	100%	3.76	0.44
11	Discussions in the comments section help me gain new perspectives about the topic.	100%	3.48	0.51
12	Face-to-face discussions about the topics shared on Instagram help clear up any misunderstandings that I may have.	86%	3.41	0.73
13	After the class ended, I continued to think about my written comments.	86%	3.00	0.53
14	I still like to play Instagram stories even after the class has ended.	86%	3.14	0.69
15	After the class, Instagram helped me to think about the discussions held in the classroom.	97%	3.28	0.53
16	After the class, the Instagram posts helped me reconsider and develop my ideas and thoughts.	100%	3.48	0.51
17	Instagram helped me to think outside the box.	93%	3.34	0.61
18	I like using Instagram in learning because it allows me to access learning materials any time during the day.	86%	3.45	0.74
19	I like using the emojis in the comments section instead of writing.	79%	3.24	0.79
20	Instagram is easy to use.	100%	3.90	0.31
21	The informal atmosphere of Instagram makes it enjoyable and more flexible to learn.	97%	3.59	0.57

The results show that most of the students indicated their strong willingness and desire to use Instagram to study inside and outside the classroom. Concepts could be learned by students through Instagram because students demonstrated their learning aptitude in the classroom. Average scores indicated that students perceived they had greater opportunities to learn *via* Instagram than in traditional courses. The results also show the students’ reflections on how Instagram helped students; students’ inclination toward the use of social media was greater than their inclination toward traditional methods of teaching. Therefore, our study showed that a combination of JiT teaching and PI allowed learners to understand the content taught, which was evident in the responses presented above.

Table 6 shows the significance and importance of the use of Instagram in teaching practice; students provided immediate and extremely positive responses regarding their level of understanding of tasks that were assigned to them through JiT teaching. The results also indicate students’ complete satisfaction regarding collaboration and motivation. Learners’ responses suggested that they strongly disliked traditional methods of assigning assignments. In addition, teachers also received immediate feedback from the students highlighting the gaps that should be addressed during teaching sessions to make them more appropriate to students’ current level of knowledge. The responses mentioned above present a picture of the usefulness of using Instagram in teaching and learning. Table 6 also illustrates the usefulness of Instagram for assigning reading before class when first using textbooks. In particular, the responses to several items (1–4) provide convincing support for the use of Instagram in the class. Nevertheless, it is apparent that JiT teaching and the use of social media captured the attention of instructors and students. The following graph shows that the learners showed keen interest in these approaches, and that teachers recognized improvement in students’ engagement in learning in the classroom. Students voluntarily showed their interest in discussing and completing tasks. According to Freyn (2017), students welcome any task that is taught with the use of social media.

**Figure 4:**
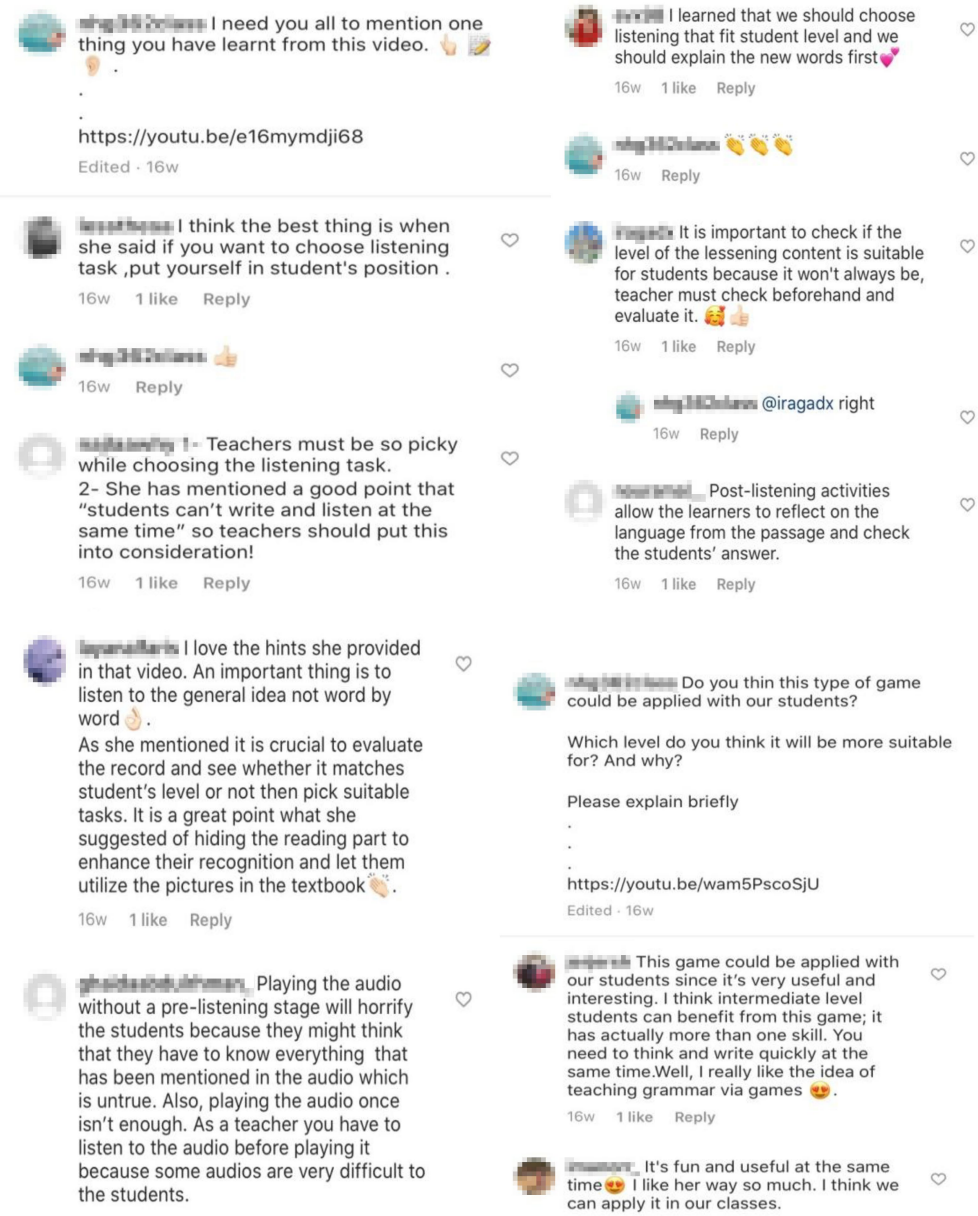
Students’ actual responses in class on Instagram.

Figure 4 is a screenshot of the responses from the private Instagram account for the course, which was used throughout the entire process of teaching (pseudonyms have been used). The responses show convincing and satisfactory feedback from the learners about the teaching and learning process. All students in the group enjoyed full liberty to communicate their ideas and to comment on the role of the teacher. Figure 4 also shows positive initiative to learn and cooperate with other learners, as well as the successful practical involvement of the students. In the discussion, the students discussed the class topics, commented on various points, and used different contextual information while completing the task in the group, which shows the successful implementation of JiT teaching in the EFL classroom.

To answer the third research question, the correlations among the four survey categories were analyzed by using SPSS (version 22). Pearson Correlation was conducted on this data. The results are shown in Table 7.

**Table 7:** Correlations among the categories of the questionnaire.

		Pre-class teaching	Active learning	Reflection
Active learning	Pearson Correlation	0.267		
	Sig. (2-tailed)	0.178		
Reflection	Pearson Correlation	0.747^**^	0.518^**^	
	Sig. (2-tailed)	0.000	0.006	
Usability and flexibility of Instagram	Pearson Correlation	0.111	0.456^*^	0.373
	Sig. (2-tailed)	0.582	0.017	0.055

* Correlation is significant at the 0.05 level (2-tailed).

** Correlation is significant at the 0.01 level (2-tailed).

Table 7 shows a significant, positive correlation between the four categories. Pre-class teaching had a small correlation with active learning (*r* = 0.267). On the other hand, Pre-class teaching had a large correlation with reflection and usability and flexibility of Instagram (*r* = 0.747, 0.582, respectively). Active learning had a medium correlation with the usability and flexibility of Instagram (*r* = 0.456). Similarly, reflection had a medium correlation with the usability and flexibility of Instagram (*r* = 0.373). To summarize, the results yielded positive correlations between all four categories.

### Focus group responses

Semi-structured focus group interviews were also conducted to obtain the following responses from students, such as descriptions of their own learning experiences in terms of motivation and collaboration; students were asked to compare their past experiences with the lecture-based teaching approach to the current semester in which Instagram was integrated into the learning process and used as a communication tool and learning platform for class topics. Finally, the students were asked which features of Instagram they liked the most and why.

### Time and effort (flexibility)

Most of the students said that Instagram saved them time and effort. It helped motivate them and relieved tension compared to past experiences. Since students used Instagram every day, it was easy for them to use because they knew how to navigate the application. Instagram was useful for acquiring knowledge quickly using the five senses. Participating and leaving comments on posts did not take much time, so it was a great way to effortlessly participate in discussion. Usually, in lectures, not all students can give their opinions because of the time limitations or their current mood; however, on Instagram, students are free to comment whenever they like, with the flexibility to keep or delete their comments.

### Learning outcomes (effectiveness)

Students indicated that using social media, especially Instagram was very helpful for learning another language. Teachers gained some experience in how to use social media in class effectively, gathering information on how they could teach properly and could access wider references through different forms of media. Teachers exerted less time and effort with Instagram than with lecture-based teaching. The learning experience was truly effective because of its practicality, and the outcomes were more effective than those with traditional teaching approaches.

One of the features that students liked on Instagram was their ability to see their friends’ comments and their responses in addition to the original post. In the learning process, teachers and students were able to develop their thoughts by typing their opinions about the videos that they posted on Instagram. Additionally, teachers were able to predict which approach would be good for learners and how to facilitate English language learning for learners and make it enjoyable.

### Learner autonomy (motivation)

Instagram was a motivating tool for students, encouraging learners’ autonomy by giving them the ability to interact from anywhere. All respondents (students and teachers) noted that using social media in teaching and learning was something new and enjoyable for them that simplified learning. In the past, teachers had been the only source of learning, but this different use of social media allowed students to learn at their own pace encouraging autonomous learning and making room for fun and interaction with other enthusiasts who posted about useful topics. Furthermore, any new learning material and necessary information could be provided by teachers with posts on Instagram.

One student, Sarah, said, “*We had fun seeing videos… different topics in language and interacting with each other’s and with the teacher.*”

Another student, Lyla, noted, “*Instagram is an application that we use frequently… why not make use of it in school … it will help us and benefit us especially… it does not take much time and is not boring.*”

The above learner interview excerpts indicate that the learners viewed the use of Instagram in the experiment to be more interesting and fruitful than traditional teaching methods for enhancing learners’ ability to discuss their views and viewpoints. Mazur (1997b) argued that the effectiveness of practicing PI in a classroom depends upon the quality of the technique. In this study, PI and JiT teaching were viewed as welcome instruments to be used within the classroom by the learners. PI focuses on the previous knowledge of the learners, and Instagram is available everywhere and can be used by students at any time to read materials to gain knowledge directly, which facilitates pre-class reading. This practice also encourages learners to ask more questions during classroom interaction with teachers.

## Discussion

This paper focused on the use of JiT teaching and PI for students’ achievement and learning in combination with traditional ways of teaching. Encouraging student engagement can be a challenge. In this research, the authors aimed to identify significant characteristics related to student engagement. According to the results, activating students’ prior knowledge before class allows students to be more relaxed and increases their confidence, which in turn enhances their motivation to participate in class discussions.

While JiT teaching involves activities outside of the classroom, PI is applied inside the classroom; a combination of these two methods was very successful with the implementation of Instagram as a communicational tool. The authors conclude that JiT teaching and PI are very helpful in improving the in-depth learning of the selected topic and that Instagram is an effective learning tool to improve learners’ understanding. A key to the success of the implementation of JiT teaching is the development of a set of effective questions to be posted for students to answer before class. JiT teaching allows instructors to gather information about students’ understanding of the course and tailor activities to meet students’ actual learning needs. The study highlights that through the use of PI, learners and teachers obtain quicker feedback than through the use of traditional teaching methods; this more immediate feedback is more helpful for instructors, as it is provided in time to be applied in class. In addition, the study indicated that the combined JiT and PI strategy made the time spent in the classroom teaching students difficult items more pleasant and that it created a friendly environment for the learners. The results of the study presented above clearly show the importance of PI and JiT teaching and the positive role of Instagram as a valuable instrument for increasing learners’ awareness and interest. In addition, the study also highlighted that PI and JiT teaching greatly contributed to assisting learners in providing correct responses and increased their motivation to learn through the creation of a friendly environment. Although JiT teaching was a new approach for the participants in this study, the participants still completed the activities and showed a keen interest in learning with the latest technology. In sum, the study shows that Instagram and JiT teaching are tools that have transformed traditional ways of teaching and student-teacher interaction in the learning process.

The relationships between the four categories reveal a significant correlation among the four categories. Pre-class teaching had a large correlation with reflection and usability, and flexibility of Instagram. In addition, pre-class teaching had a small correlation with active learning. Also, active learning and reflection had a medium correlation with the usability and flexibility of Instagram. In general, the results indicate positive correlations among the four categories.

## Conclusion

The purpose of this study was to investigate whether the use of Just-in-Time teaching and peer instruction enhances students’ achievement in a second language. It also aimed to explore students’ perceptions of using Instagram to improve discussion, collaboration, and motivation. Findings indicated that activating students’ prior/background knowledge before class allows students to be more relaxed and increases their confidence, which in turn enhances their motivation to participate in class discussion. Positive correlations between pre-class teaching, active learning, reflection, usability, and flexibility of Instagram were also found. This study therefore concludes that JiT teaching and PI are very helpful in improving students’ in-depth learning of a selected topic, and that Instagram is an effective learning tool to improve learners’ motivation and understanding.

### Pedagogical implications

The results of this study have practical implications for pedagogy in teaching English as a foreign language. Just-in-Time teaching can be used to augment in-class teaching by giving students enriching assignments that can be done at home/outside of class and/or in groups. For the successful implementation of JiT teaching in English language teaching and learning, this study recommends that EFL instructors clearly describe to students how JiT teaching works, its aim, and everyone’s respective roles to make it a success. Additionally, EFL instructors need to continually monitor and evaluate students’ learning. By observing students’ progress with JiT teaching practitioners can make modifications throughout the process to better meet students’ diverse learning needs.

## Data availability statement

The raw data supporting the conclusions of this article will be made available by the authors, without undue reservation.

## Ethics statement

Ethical review and approval was not required for the study on human participants in accordance with the local legislation and institutional requirements. Written informed consent from the patients/participants OR patients/participants legal guardian/next of kin was not required to participate in this study in accordance with the national legislation and the institutional requirements.

## Author contributions

HA has contributed in the write-up of the introductory section and literature of the study. MA did the analysis, and discussion section of the study. In addition, he did the final proofreading and editing before the submission. RA collected the data collection and overall supervision of the study. SC proofread the final paper edits and restructured and rewrote sections of the paper. All authors contributed to the article and approved the submitted version.

## Funding

This work was supported by Researchers Supporting Project Number (RSP-2021/251) King Saud University, Riyadh, Saudi Arabia.

## Conflict of interest

The authors declare that the research was conducted in the absence of any commercial or financial relationships that could be construed as a potential conflict of interest.

## Publisher’s note

All claims expressed in this article are solely those of the authors and do not necessarily represent those of their affiliated organizations, or those of the publisher, the editors and the reviewers. Any product that may be evaluated in this article, or claim that may be made by its manufacturer, is not guaranteed or endorsed by the publisher.
